# Supplementary material for: Probiotic Supplementation Improves Gut Microbiota in Chronic Metabolic and Cardio-Cerebrovascular Diseases Among Chinese Adults over 60: Study Using Cross-Sectional and Longitudinal Cohorts
Source: Microorganisms. 2025 Jun 27;13(7):1507. doi: 10.3390/microorganisms13071507 (PMC12299297; doi:10.3390/microorganisms13071507)
Supplement: Supplementary file 1 [file microorganisms-13-01507-s001.zip › Supplementary_figure.pdf]

Supplmentary Materials

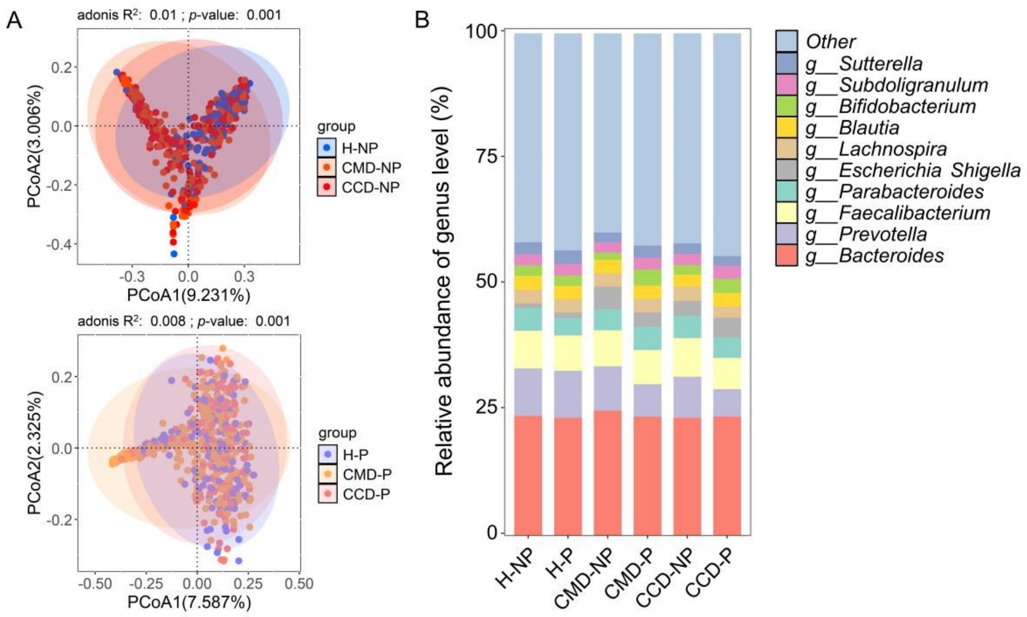

Supplementary Figure S1. Analysis of the structure and composition of gut microbiota in the cohort 1. (A) β-Diversity of the bacterial microbiota in NP and P groups at the ASV level. PCoA visualization employing Bray-Curtis distance. Statistical evaluation of inter-group differences was implemented through adonis tests (999 iterations), with R^2 values quantifying explained variance and *p*-values denoting significance displayed in panels. (C) Global composition of bacterial microbiota at the genus level in six subgroups. Only the top 10 taxa are presented in the graph.

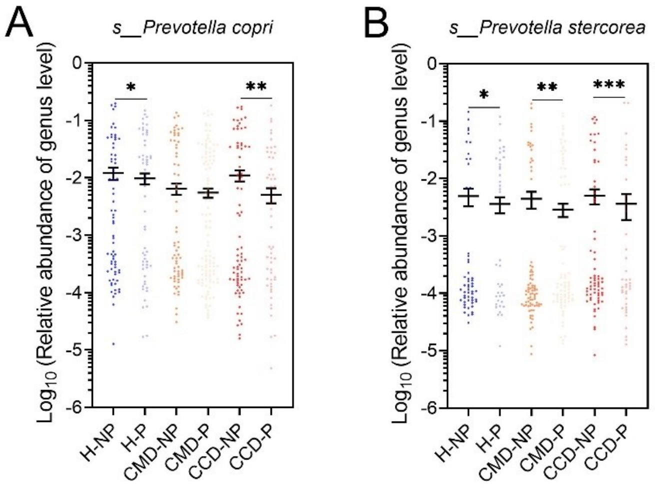

Supplementary Figure S2. Species-level changes of *Prevotella* in the cohort 1. A. Changes in the relative abundance of *Prevotella copri* in the intestines of six subgroups. B. Changes in the relative abundance of *Prevotella stercorea* in the intestines of six subgroups. Mann-Whitney U test was used for statistical analysis, *, $p < 0.05$, **, $p < 0.01$, ***, $p < 0.001$.

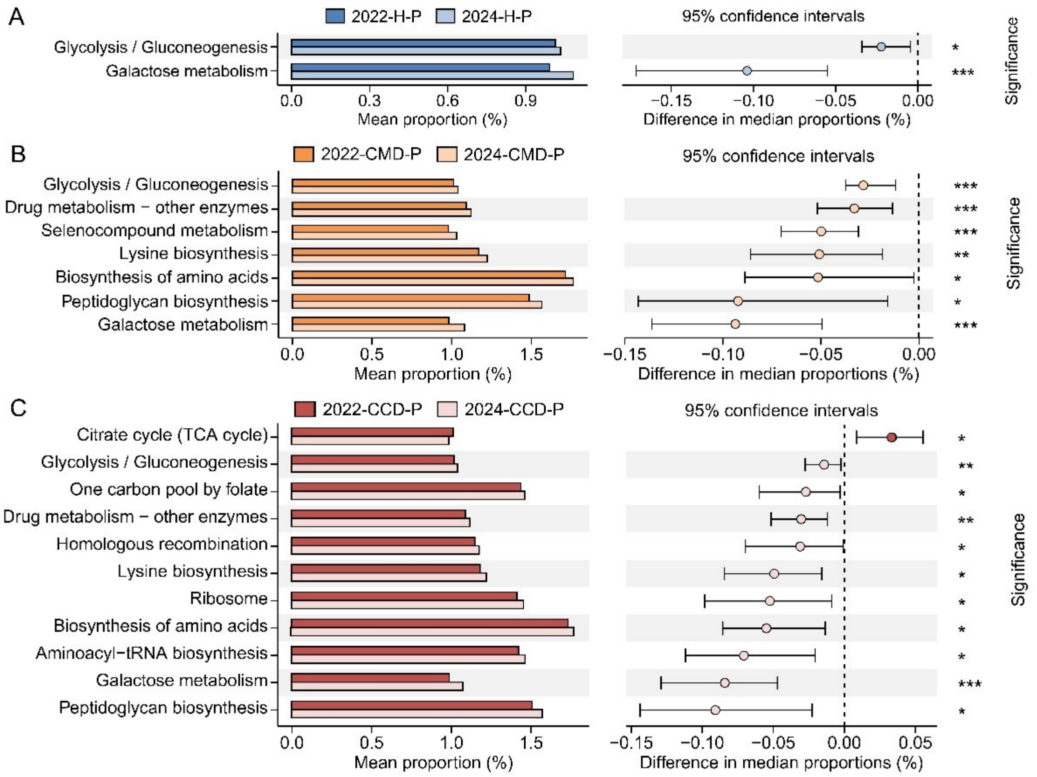

Supplementary Figure S3. The effects of long-term probiotic supplementation on KEGG metabolic pathways across the groups. (A) The effect of long-term probiotic supplementation on KEGG metabolic pathways in the gut microbiota of the H-P group. (B) The effect of long-term probiotic supplementation on KEGG metabolic pathways in the gut microbiota of the CMD-P group. (C) The effect of long-term probiotic supplementation on KEGG metabolic pathways in the gut microbiota of the CCD-P group. The KEGG metabolic pathways of the intestinal microbiota were predicted using the PICRUSt2 tool. The minimum alignment rate was set to 0.8. The mean proportion and the difference in the mean proportions for pathways showing a significant difference in abundance are shown. Statistical tests for differences in data were performed using the Mann-Whitney U test, and *p*-values were adjusted using the false discovery rate (FDR) method. *, $p < 0.05$, **, $p < 0.01$, ***, $p < 0.001$.
